# The relationships between negative emotions and latent classes of smartphone addiction

**DOI:** 10.1371/journal.pone.0248555

**Published:** 2021-03-15

**Authors:** Heng Yue, Xuemin Zhang, Junfang Sun, Min Liu, Cuiyun Li, Hugejiletu Bao

**Affiliations:** 1 School of Psychology, Inner Mongolia Normal University, Hohhot, Inner Mongolia, China; 2 College of Physical Education, Inner Mongolia Normal University, Saihan District, Hohhot, Inner Mongolia, China; Sun Yat-sen University, CHINA

## Abstract

The relationships between negative emotions and smartphone addiction has been tested through the literature. However, most of the studies applied variable-centered approaches. The heterogeneity of smartphone addiction severity has not been examined for the associations with negative emotion variables. The purposes of the present study is to explore the latent classes of smartphone addiction and analyze the relationships between depression, social anxiety and boredom and these subgroups. The Smartphone Addiction Scale-Short Version (SAS-SV) and three negative emotion scales were employed to conduct a survey of 539 college students. Mplus8.3 software was applied to perform the latent class analysis (LCA) based on the smartphone addiction symptom ratings. ANOVA and multinomial logistic regression were used to explore the differences among these latent categories and the associations between these subgroups and negative emotion variables. Results demonstrated that Negative emotional variables were significantly correlated with smartphone addiction proneness. Based on their scores on the Smartphone Addiction Scale, smartphone users were divided into three latent classes: low risk class, moderate class and high risk class. Women were more likely to be classified in the high-risk class. The severity of depression and boredom was able to predict the membership of the latent class effectively; while social anxiety failed to do this in the high risk class.

## 1. Introduction

Smartphones are widely used across the world and have been an indispensable part of our daily life [1, 2]. As one of the great achievements of science and technology development, smartphones provide many useful functions, such as calling, messaging, online shopping and entertainment. With the help of these intelligent devices, people’s interpersonal relationships and daily life quality were improved; and the productivity in the workplace was enhanced as well [1, 2]. Smartphones are highly portable and can be accessed almost anywhere and at any time, moreover, there are considerable interesting applications (Apps) can be installed on this device. With these priorities, smartphones have been the most preferred and frequently used communication instruments in modern society, thus, addiction of smartphones by individuals especially adolescents have emerged as a significant social issue [3, 4].

Smartphone addiction is defined as excessive use with associated functional impairment and resulting symptoms found in substance use disorders such as withdrawal and tolerance [5, 6]. Researchers have found that smartphone addiction is associated with many physical and mental health problems. Individuals who addicted to smartphones may experience higher depression severity, bodily pain and daytime sleepiness [7]. Excessive screen time is related to lower self-esteem and the risk of premature cognitive decline [8]. Evidence also suggests that smartphone use has a negative association with peer relations and family relations [9]. Participants who spend more time on their smartphones report lower well-being and life satisfaction [10]. Due to the fact that smartphone addiction can lead to so many detrimental consequences, therefore, it is indispensable for scholars to update their perspectives and research methods to explore the risk factors that place individuals at risk of smartphone addiction. This will contribute to providing more knowledge, better understanding of smartphone addiction, and offering valuable suggestions for tackling this problem.

Negative emotions such as depression, social anxiety and boredom are important antecedents of smartphone addiction. The associations between depression, social anxiety, boredom and smartphone addiction have been tested by many previous studies. Depression is conceptualized as a common mental disorder which not only impacts the way people feel, think and behave, but also impairs individuals’ social or occupational functions [11]. Researchers have suggested that depression is a positive predictor of smartphone addiction, and individuals who suffer from depression symptoms tend to use smartphone excessively to eliminate their psychological distress [12, 13]. Empirical researches have found longitudinal and bidirectional relationships between smartphone addiction and depressive symptoms [14, 15]. Therefore, depression is an important variable in the study of smartphone addiction. Social anxiety is a type of anxiety that results from the prospect or presence of personal valuation in real or imagined social situations [16]. Studies have indicated that social anxiety is positively associated with smartphone addiction, and the reason lies in the fact that smartphone may serve as an avoidance strategy of face-to-face interactions for individuals who experience social anxiety [17, 18]. Thus, individuals with high social anxiety may tend to spend more time on smartphones than face-to-face communication. Boredom is an aversive experience of weariness, restlessness and constraint, associated with both monotonous environments and individual characteristics, such as inattention [19]. Previous studies have confirmed that boredom is positively correlated with smartphone addiction, because people often use smartphones to overcome boredom and achieve physiological arousal or positive internal states, and this will in turn lead to smartphone addiction [20, 21].

According to the literature review, most of the recent studies have investigated the associations between smartphone addiction and psychopathology with a variable-centered approach. However, some scholars argue that the variable-centered approach does no allow researchers to explore the relationships between the level of variable for a specific person and the levels of other variables, and this method does not provide any information about the person-specific psychological processes and behaviors [22]. Consequently, the heterogeneity of a target group cannot be distinguished precisely and effectively. Therefore, in the studying regarding smartphone addiction, more attention needs to be paid to the differences of the participants. Furthermore, a recent study has suggested that there exists a discrepancy between the participants’ views about the severity levels of their smartphone usage and their real scores on the smartphone addiction scale. Nearly one-fifth (18.3%) of the high scores did not regard their smartphone use as problematic; compared with this, 55.1% of the low scorers reported that their problematic smartphone use [23]. The result of this study is similar to the problems shown in the diagnosis of mental disorders. At present, mental disorders are often divided into different types only based on the number of diagnostic criteria that individuals meet. However, there are many limitations of categorical classifications; and this practice has been questioned by many empirical researches [24]. In fact, the main problem is that each category of mental disorder is not a completely discrete entity with absolute boundaries dividing it from other mental disorders or from no mental disorder [25]. Therefore, model-based approaches are required to estimate and distinguish the latent heterogeneous classes of the target group [26].

Latent Class Analysis (LCA) is a person-centered approach. It clusters individuals by drawing on their shared characteristics and divides them into mutually exclusive classes [22]. LCA is a model-based approach, it provides a set of fit indices for estimating the goodness of the proposed latent class model represents the data [27]. This makes the results of LCA more objective and precise. LCA has also been used to identify prototypical configurations or typologies of individuals based on participants’ responses to the observed variables. Because all items of an assessment tool are employed to identify the latent classes, therefore, one latent class categorized by LCA includes individuals with the similar symptoms and similar severity of the specific disorder [28]. Compared with variable-centered analyses, such as correlation analysis, LCA focuses on individuals, LCA can take full advantage of the available information provided by the assessment tools and yield a more reasonable classification scheme. In some studies, LCA has been employed to establish scientific cut-off points for assessment tools as well, such as online social networking addiction, Bergen social media addiction scale (BSMAS), and the ten-item internet gaming disorder test (IGDT-10) [28–30]. Given that when the clinical interviews are absent, LCA is considered as the best method for understanding heterogeneity within diagnostic classes [28, 31], an increasing number of studies are conducted by employing this approach.

Two theories may contribute to explaining and understanding the relationships between negative emotional variables and the latent classes of smartphone addiction. According to compensatory internet use theory (CIUT), technologies or mediums such as the internet and smartphone are applied as a means to alleviate people’s negative emotion, and satisfy their social needs [32]. From the perspective of this theory, negative emotion can be regarded as the antecedent of smartphone usage and addiction, and excessive smartphone use is deemed as the compensatory behavior targeted at regulating negative emotions [33]. The information theory of emotion posits that emotion is determined by the difference between available and necessary information, and negative emotions are generated by the shortage of the available information [34]. The more the available information lacks, the more severe individual’s negative emotion becomes. An individual will experience negative emotions when his (or her) needs are not being met (lacking of the available information), at the moment, virtual resources such as smartphones will be got as an alternative [35]. In addition, previous studies have demonstrated that the individual’s needs can be generalized. For instance, attachment anxiety has an effect on hoarding behavior [36]; stress induces more food intake and increases the risk of drug abuse and relapse [37]; social anxiety has an impact on individual’s tobacco and alcohol use [38]. Therefore, when a person experiences negative emotions, he (or she) is likely to generalize the requirement of the information. Smartphone, which contains various information and can be available almost at any time and anywhere, become the first choice and optimal instrument to be used.

From what has been mentioned above, the relationships between depression, social anxiety, boredom and smartphone addiction are required to be reconfirmed in a person-centered approach. Therefore, it is imperative to conduct the latent class analysis to uncover the latent subgroups of the participants based on their scores of smartphone addiction scale, and examine the associations between negative emotional variables and the latent types of smartphone addiction.

In the present study, latent class analysis (LCA) is adopted to identify the latent types of smartphone addiction for college students. In addition, the relationships between these latent types and negative emotions will be analyzed. This study is important in understanding the heterogeneity of smartphone addicts and associated psychopathology.

## 2. Method

### 2.1 Participants

A total of 539 college students were enrolled in the present study. In all, 570 questionnaires were sent out and 554 questionnaires were returned. However, 15 questionnaires were excluded because these participants failed to complete the questionnaires. Ultimately, the data from the remaining 539 questionnaires were used for the final analysis. Among the participants, there were 312 men and 227 women. The mean age of the participants was 20.44 ± 1.41 years.

### 2.2 Measurements

#### 2.2.1 Patient Health Questionnaire (PHQ-9)

Depressive symptoms were assessed by the PHQ-9 [39]. This instrument contained 9 items, each item was rated on a 4-point Likert scale (0 = not at all, 3 = nearly every day). The higher sum scores on the PHQ-9 indicated greater severity of depression. In the present study, the Cronbach’s alpha coefficient of this scale was 0.94.

#### 2.2.2 Social anxiety scale

Social anxiety was measured by the social anxiety scale [40]. This scale consisted of 6 items. Participants answered each item on a 5-point scale, from “strongly disagree” (tagged with 1) to “strongly agree” (tagged with 5), with high scores indicating higher degree of social anxiety. In the present study, the Cronbach’s alpha coefficient of this scale was 0.82.

#### 2.2.3 Short boredom proneness scale (SBPS)

Boredom was evaluated with the SBPS [41]. This scale had 8 items, and participants were asked to respond on a 7-point scale (from 1 = “strongly disagree” to 7 = “strongly agree”). The higher the score of respondent was, the higher the level of individual’s boredom proneness. In the present study, the Cronbach’s alpha coefficient of this scale was 0.93.

#### 2.2.4 Smartphone addiction scale—short version (SAS-SV)

Smartphone addiction was assessed by SAS-SV [42]. This scale contained 10 items, each item was rated on a 6-point scale (from 1 = “strongly disagree” to 7 = “strongly agree”). High sum scores obtained from the SAS-SV indicated a high risk of the smartphone addiction. In the present study, the Cronbach’s alpha coefficient of this scale was 0.92.

### 2.3 Procedure

This study was approved by the College of Psychology Inner Mongolia Normal University. The data were collected in the classrooms of five universities in Inner Mongolia. Before distributing the questionnaires, all the participants were informed that the data were used merely for scientific research, they were not forced to participant in this study; their responses were anonymous; they could quit at any time they would like and this would not affect their life and their final academic performance. Finally, after obtaining the consent from the participants and their teachers, questionnaires were only distributed to the individuals who agreed to participate in this study.

### 2.4 Statistical analysis

Microsoft Excel 2016 was used to input the data. SPSS 22.0 was used to transform the data file, and conduct descriptive statistics, correlation and multiple logistic regression analysis. Mplus8.3 software was employed to perform LCA based on the items of SAS-SV. Akaike information criterion (AIC), Bayesian information criterion (BIC), adjusted Bayesian information criterion (aBIC), Entropy, Lo-Mendell-Rubin (LMR) and Bootstrapped likelihood ratio test (BLRT) were obtained to determine the optimal number of latent classes. For AIC, BIC and aBIC, smaller values indicated a better-fitting model. Entropy is an index for estimating the quality of class assignment; a higher entropy indicated a greater precision of classification [43, 44]. Both the LMR test and BLRT yielded a p value. A significant p value suggested that the *k*-class model was better than the *k-1*-class model [45].

## 3. Results

### 3.1 Descriptive statistics

Table 1 showed the means, standard deviations, and Pearson correlation coefficients of the study variables (n = 539). As seen, the total scores of depression averaged 17.23 (SD = 6.14). Social anxiety summed scores averaged 17.76 (SD = 4.88). Boredom total scores averaged 30.40 (SD = 10.58). The sum of smartphone addiction items averaged 35.34 (SD = 10.319). Depression, social anxiety, boredom had significant and positive correlation with smartphone addiction.

**Table 1:** Means, standard deviations, and Pearson correlation coefficients of the study variables (n = 539).

Variables	Mean ± SD	Depression	Social anxiety	Boredom	Smartphone addiction
Depression	17.23 ± 6.14	1			
Social anxiety	17.76 ± 4.88	0.35**	1		
Boredom	30.40 ± 10.58	0.62**	0.55**	1	
Smartphone addiction	35.34 ± 10.32	0.46**	0.38**	0.62**	1

Note: ** P < 0.01.

### 3.2 Primary LCA results

The 10 items of the smartphone addiction scale was employed in LCA. The models were continually tested starting with one latent class, and the number of classes was progressively increased until no significant improvement in model fit was achieved. The results were shown in Table 2. With the increasing of the number of latent classes, values of AIC, BIC and aBIC were gradually decreased. The value of Entropy achieved the maximum when the number of latent classes was three. The p values yielded by LMR test and BLRT indicated that the model with three latent classes fitted best. Therefore, the 3-class model was considered as the final model. The probability of correct classification was 95% for Class 1, 97% for Class 2, and 96% for Class 3 respectively, and furtherly, this suggested that it was reasonable to divide participants into three latent classes.

**Table 2:** Latent profile analysis model comparisons.

Latent class	K	AIC	BIC	aBIC	Entropy	LMR	BLRT
1	20	18694.30	18780.10	18716.61	--	--	--
2	31	17016.73	17149.72	17051.31	0.88	<0.01	<0.01
3	42	16321.51	16501.68	16368.35	0.91	<0.01	<0.01
4	53	16082.53	16309.88	16141.64	0.87	0.09	<0.01
5	64	15844.97	16119.51	15916.35	0.88	0.10	<0.01

**Fig 1:**
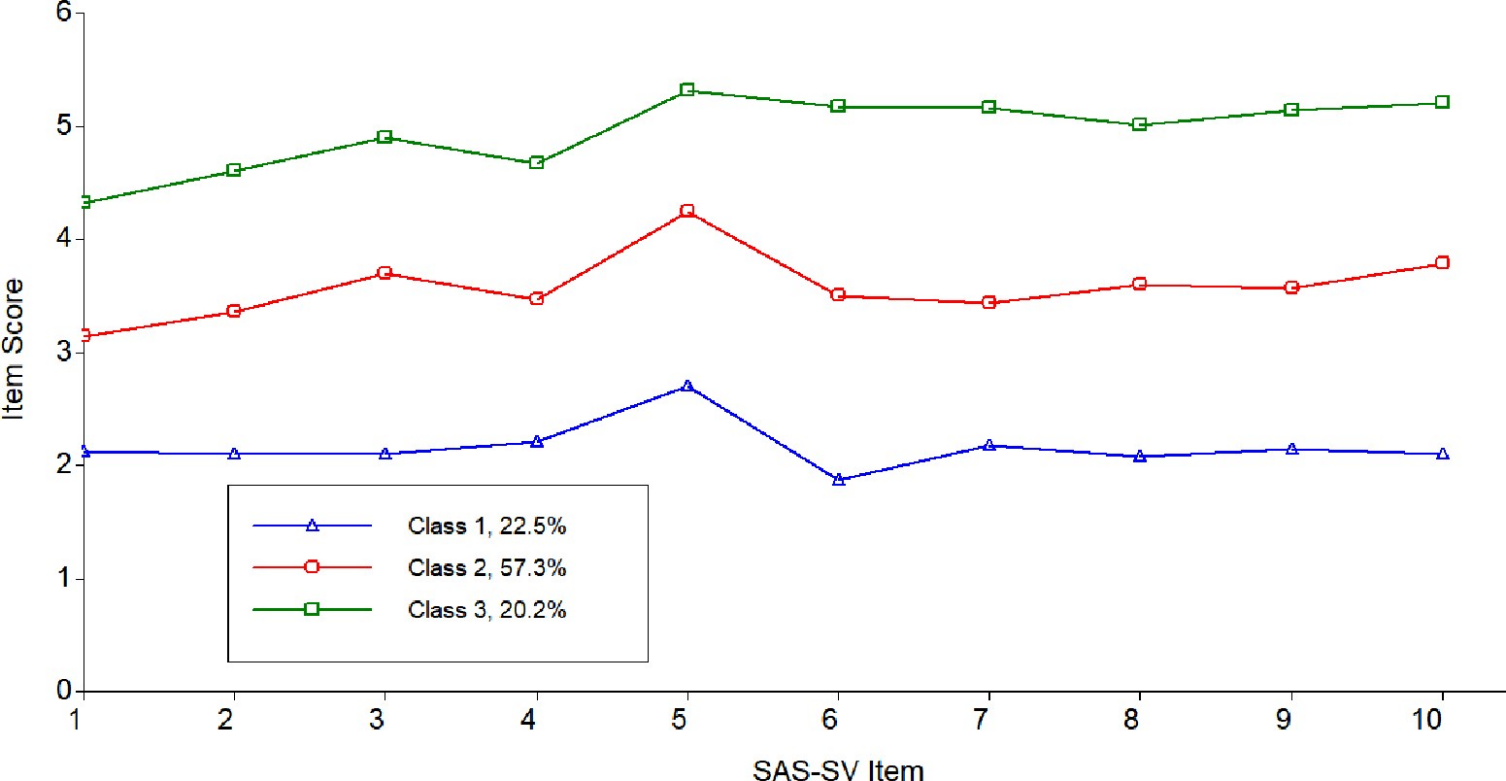
Latent classes from the three-class solution.

Based on the severity ratings, a graphical illustration of the three levels of smartphone addiction was displayed in Fig 1. As seen, the differences among the three latent classes were obvious. Class 1 appears to represent individuals with low levels of smartphone addiction. This class included 119 individuals, accounted for about 22.5% of the participants. Because individuals in this class had relatively low scores, this class was named “low risk class”. Class 2 consisted of 313 individuals, accounted for close to 57.3% of the participants. The scores of the participants in this class were higher than Class 1, while lower than Class 3. Therefore, this class was named “moderate class”. Class 3 represented individuals with the highest risk level for smartphone addiction. The name of this class was “high risk class”. This class contained 107 individuals, accounted for approximately 20.2% of the entire sample.

### 3.3 Comparisons of negative emotions across latent classes

ANOVA was performed to explore the differences of negative emotions across latent classes. These findings were shown in Table 3. Significant differences in depression, social anxiety and boredom were confirmed across different latent classes. In post hoc analyses, scores of negative emotions of high risk class were higher than low risk and moderate class, and that of moderate class was higher than low class.

**Table 3:** Comparisons of negative emotions across latent classes (n = 539).

Variables	Low risk class	Moderate class	High risk class	*F*	Post hoc
Depression	13.92 ± 4.47	16.86 ± 5.22	21.97 ± 7.30	60.85**	C3>C2>C1
Social anxiety	15.20 ± 5.04	17.89 ± 4.33	20.19 ± 4.89	33.31**	C3>C2>C1
Boredom	22.52 ± 9.51	29.82 ± 7.98	40.84 ± 9.93	125.19**	C3>C2>C1
Smartphone addiction	21.32 ± 5.24	35.78 ± 4.28	49.64 ± 5.30	1018.44**	C3>C2>C1

Note: ** *P* < 0.01. C1, Class1. C2, Class2. C3, Class 3.

### 3.4 Effects of demographic and negative emotional variables on latent classes

Multinomial logistic regression was applied to explore the effects of demographic and negative emotion variables on latent classes. The results were displayed in Table 4. As seen, gender (woman) had a marginal significant effect on predicting the membership of high risk class (*P* < 0.06). Boredom was a valid predictor in the classification of the moderate and high risk class. Each added point for boredom tendency was associated with 7% and 23% increased likelihood of being in the moderate and high risk class. Depression predicted the membership of the moderate and high risk class at a marginal significant level (*P* < 0.07) and a significant level (*P* < 0.01) respectively. For every one-point increased in depression tendency, the odds of being the membership of moderate and high risk class would increase 5% and 10%. Social anxiety predicted the membership of the moderate class with a marginal significant effect (*P* < 0.07), whereas it failed to do this in the high risk class.

**Table 4:** Results of multinomial logistic regression (using the low risk class as the reference class).

	Moderate class					High risk class				
Variables	*B*	*SE*	*Wald*	*Exp(B)*	*p*	*B*	*SE*	*Wald*	*Exp(B)*	*p*
Gender	0.16	0.24	0.45	1.18	0.50	0.64	0.40	3.52	1.89	0.06
Age	0.02	0.09	0.05	1.02	0.81	0.11	0.12	0.80	1.11	0.37
Depression	0.05	0.03	3.35	1.05	0.07	0.10	0.04	7.24	1.10	0.01
Social anxiety	0.05	0.03	3.31	1.05	0.07	0.01	0.04	0.04	1.01	0.83
Boredom	0.07	0.02	15.74	1.07	0.00	0.21	0.03	63.18	1.23	0.00

## 4. Discussion

Based on a person-centered perspective, and by adopting SAS-SV, this study explored the latent classes of smartphone addiction. Moreover, the relationships between these latent types and negative emotions such as depression, social anxiety and boredom were analyzed. The main findings and implications of this study are presented as follows:

According to their responses on items of SAS-SV, participants were divided into three latent classes: low risk class, moderate class and high risk class. This result is similar to the findings of previous studies [5, 46]. When smartphone addiction is considered solely, the subgroups identified by LCA are consistent. However, some scholars have also indicated that problematic smartphone use combined with problematic internet use can be categorized into three, four or six latent classes [47, 48]. The differences in the number of the latent classes may result from the inconsistency of the assessment instruments adopted or the discrepancy of the participants enrolled. For the former reason, the following explanation may be helpful. Previous studies have suggested that compared with smartphone addiction, internet addiction is a more comprehensive concept, because most of the smartphone functions or applications such as social media or browsing the web rely on an access to the internet, thus, smartphone usage represents nothing more than internet use [49]. In addition, smartphone represents only one way to access the internet, other approaches such as desktop computers, digital tablets or video game devices may not be considered when participants respond to the questionnaires. According to Media Dependency Theory, goals are important antecedent motivations within individual’s dependency relations with the media [50]. If people find the resources provided by the media favorable for achieving various goals, they may develop dependency relationships with the media [51]. Given the fact that internet can provide various approaches that contain more media resources than smartphones, therefore, internet addiction may have more or at least equal number of underlying groups than smartphone addiction. For the latter reason, individuals’ characteristics may be a possible explanation. Participants enrolled in the latent class analysis of both internet and smartphone addiction are adolescents. Adolescence is a vulnerable stage in human development since it represents a transition from childhood to physical, psychological and social maturity [52]. Due to the immaturity of their cerebral cortex, adolescents have relatively higher levels of impulsivity and lower levels of self-control [53]. Compared with adults, their emotions are more unstable, and more susceptible to the context; meanwhile, compared with children, adolescents’ responses to the life events are more intense [54]. This may drive and intensify them to seek extraneous experience [54]. From what has been mentioned above, adolescents’ internet usage habits may easily be influenced by various factors, and they are separated in different latent classes of internet addiction.

The results of ANOVA suggested that different latent categories had their unique negative-emotion characteristics. Scores of participants of moderate and high-risk class on negative emotional variables were significantly higher than that of low risk class. The differences of the scores of these latent categories can be found in some empirical researches. On one hand, researchers have confirmed that depression, social anxiety and boredom are major risk factors for smartphone addiction [14, 17, 21], the levels of individuals’ negative emotions are associated with the severities of their smartphone addiction symptoms. On the other hand, studies have also indicated that smartphone addiction can bring about depression and social anxiety [55, 56], leisure boredom is considered as a character of one addictive behavior that is related to smartphone addiction [57]. Therefore, when people are classified into a higher risk subgroup, their scores of negative emotions tend to be high as well.

Logistic regression analysis confirmed that women were more likely to be identified as a member of the high-risk class. Nevertheless, gender could not distinguish the membership of moderate and low risk class effectively. This indicates that gender differences in smartphone addiction can be observed only in the high-risk level. Researchers have found that negative emotion is correlated to smartphone addiction [58]. In addition, since women express more intensive adverse experiences than men do when they are facing negative stimuli; and they tend to estimate negative events as more negative [59]. According to compensatory internet use theory, smartphone is applied as a means to alleviate people’s negative emotion [32]. Moreover, according to the information theory of emotion [34], emotion is determined by the difference between available and necessary information, and negative emotions are generated by the shortage of the available information. The more the available information lacks, the more severe individual’s negative emotion becomes. When individual’s negative experience is at a low or moderate level, it may be effectively eliminated by using smartphones. However, for people with a high-level negative emotion, increased amount and intensity of smartphone use are required to obtain the available information (or the substitute information) to compensate their psychological distress. Given women are more vulnerable to negative emotions; in this case, they are more likely to be distributed to the high-risk class.

Boredom was able to predicted the membership of the three latent classes significantly. Boredom is characterized as mild in negative valence, low in arousal, lacking of perceived meaningfulness and involving low attention given to situations and tasks [60]. Therefore, boredom creates a sensation seeking state that motivates individuals to explore novel experiences that can elicit different feelings [61]. From the perspective of the compensatory internet use theory and the information theory of emotion, boredom can be regarded as the requirement for novel experiences. In this case, smartphone, which offers various functions and can be used almost anywhere and anytime, becomes an important and effective instrument for those boredom-prone people to obtain different experiences. Previous study also suggests that the hedonic and eudemonic functions of the smartphone may significantly alleviate people’s perceived boredom in free time [62]. Thus, boredom proneness drives their addictive tendency and predicts the membership of smartphone addiction [63, 64].

In the moderate class, compared with the low risk class, the predicting effect of social anxiety was significant, and the effect of depression reached a marginal significant level (P < 0.07). In the high-risk class, the effect of depression was significant, while social anxiety failed to predict the membership of the high-risk class. Some possible explanations are listed below.

In terms of depression, previous study suggests that smartphone usage may be considered as an experiential avoidance strategy to divert aversive emotional content [35]. This may be because smartphone offers a virtual environment, in which individuals can not only find some available information or substitute resources, but also freely share and express their negative affects. When individuals with depress tendencies pick up their smartphones, online social support will fulfill their psychological requirements and alleviate their psychological distress [65, 66]. Moreover, they may experience flow during their usage time [67], positive emotion may reinforce people’s addiction tendencies. In this way, according to the compensatory internet use theory and the information theory of emotion, smartphones help people to cope with their psychological distress and strengthen their addictive smartphone use. Consequently, the more severe their depression symptoms are, the more urgent their requirements to alleviate their negative experiences will be, correspondingly, the higher the levels of their smartphone addiction will be as well.

As far as social anxiety is concerned, individuals with social anxiety often have a marked or intense fear of social situations in which they may be scrutinized by others [68]. Thus, they tend to avoid social situations [69]. From the perspective of the information theory of emotion, socially anxious people lack social skills, social supports or other available information that is required when they communicate with other people; therefore, they feel embarrassed and uncomfortable during their interaction with others. Given the fact that smartphone is capable of providing various internet-based communication functions, such as instant message, social media and so on. For those social anxious people, this intelligent device serves as a good substitute for face-to-face interactions. Previous study suggests that individuals with high social anxiety were more likely to interact with others through their smartphones, and have a high tendency to smartphone addiction [70]. This fits with the compensatory internet use theory that consider smartphone is used for reducing the negative emotions. As a consequence, the more severe individuals’ social anxiety is, the higher levels of their smartphone addiction will be. However, social anxiety failed to predict the membership of the high-risk class. In fact, when added solely into the logistic regression function, social anxiety had a good effect on the membership of both moderate and high-risk classes. Whereas, the effect was weakened by the action of other variables, the most probably variable is depression. Researchers have found that social anxiety is commonly related to and co-occur with depressive symptoms [71]. Moreover, people with social anxiety are more likely to experience depression [72]. Therefore, the effect of social anxiety on predicting the membership of smartphone addiction may be weakened by the action of depression.

## 5. Conclusions

According to the symptoms of smartphone addiction, smartphone users can be divided into three latent classes: low risk class; moderate class and high-risk class. When people are distributed in a higher-risk class, their symptoms of negative emotions tend to be more severe as well. Women are more likely to be classified in the high-risk class; the severity of depression and boredom can predict the membership of the latent class effectively; while social anxiety fails to do this in the high-risk class.

## Supporting information

S1 Data (ZIP)
